# Evidence of vancomycin-resistant *Staphylococcus aureus*, multidrug-resistant *S. aureus*, and *Enterococcus faecium*-causing mastitis in Thailand and Cambodia

**DOI:** 10.14202/vetworld.2025.202-209

**Published:** 2025-01-30

**Authors:** Sambo Na, Montira Intanon, Anyaphat Srithanasuwan, Wasana Chaisri, Witaya Suriyasathaporn

**Affiliations:** 1 Master’s Degree Program in Veterinary Science, Faculty of Veterinary Medicine, Chiang Mai University, Chiang Mai, 50100, Thailand; 2 School of Veterinary Medicine, Faculty of Veterinary Medicine, Chiang Mai University, Chiang Mai, 50100, Thailand; 3 Research Center of Producing and Development of Products and Innovations for Animal Health, Chiang Mai University, Chiang Mai, 50100, Thailand; 4 Department of Animal Sciences, Wageningen University, 6708, PB Wageningen, The Netherlands; 5 Cambodia-Campuses, Asian Satellite Campuses Institute, Nagoya University, Japan

**Keywords:** antimicrobial resistance, dairy farms, mastitis, methicillin-resistant *Staphylococcus aureus*, multidrug resistance, vancomycin-resistant enterococci, vancomycin-resistant *Staphylococcus aureus*

## Abstract

**Background and Aim:**

Bovine mastitis, an inflammatory condition of the mammary gland, is a critical economic issue in the dairy industry. Antimicrobial resistance (AMR) to mastitis-causing pathogens poses a significant threat to dairy operations in Thailand and Cambodia. This study investigates the AMR of mastitis pathogens in Thailand and Cambodia. It focuses on detecting methicillin-resistant *Staphylococcus aureus*, vancomycin-resistant *S. aureus* (VRSA), and vancomycin-resistant enterococci by identifying the presence of *mecA*, *vanA*, and *vanB* genes in bacterial isolates.

**Materials and Methods:**

A total of 65 bacterial isolates (55 *S. aureus* from Thailand and 10 *Enterococcus faecium* from Thailand and Cambodia) were analyzed. Disk diffusion tests were conducted for antibiotic susceptibility, and polymerase chain reaction was employed to detect AMR genes.

**Results:**

*S. aureus* isolates showed resistance to penicillin (21.8%), tetracycline (9.1%), and gentamycin (7.3%). Three isolates were identified as multidrug-resistant (MDR), resistant to tetracycline, gentamycin, and penicillin. *E. faecium* isolates exhibited high resistance to tetracycline (100%) and penicillin (90%), with 60% classified as MDR. Phenotypic analysis identified VRSA in 11% of *S. aureus* isolates. However, *mecA*, *vanA*, and *vanB* genes were not detected in any isolate.

**Conclusion:**

Mastitis pathogens in this study pose significant AMR challenges, especially with MDR *S. aureus* and *E. faecium*, and phenotypically VRSA without the *vanA* gene. The findings highlight the need for judicious antibiotic use in dairy farms and further studies with broader sampling.

## INTRODUCTION

Bovine mastitis is an inflammatory condition affecting udder tissue in the mammary gland, resulting from physical injury or microbial infections. It is regarded as the most prevalent disease-causing economic losses in the dairy industry due to decreased milk yield and quality [1]. These pathogens that cause mastitis can be found throughout the habitats of dairy cows [2]. The microorganisms commonly isolated from cows with mastitis include Enterobacteria: *Escherichia coli* and *Klebsiella pneumoniae*; Streptococci: *Streptococcus agalactiae*, *Streptococcus dysgalactiae*, and *Streptococcus uberis*; and Staphylococci: *S. aureus* and other Staphylococci. Current mastitis treatment relies heavily on antibiotics, which is the most important reason for antibiotic use in dairy cows. However, this reliance has led to the emergence of drug-resistant strains, which threaten the viability of antibiotics for mastitis treatment [3]. This underscores the urgency of the issue and the pressing need for alternative treatment methods to combat antimicrobial resistance (AMR) in mastitis pathogens because the current situation is unsustainable and poses a significant threat to animal and public health.

According to the World Health Organization, improper treatment, particularly the misuse or overuse of antibiotics, over-prescription, and inappropriate use of antibiotics, such as their use to treat viral infections, contributes to the survival of antimicrobial-resistant bacteria, leading to the proliferation of AMR strains [4, 5]. Global antimicrobial use in food-producing animals promotes animal production [6]. It could increase by 67% between 2010 and 2030 [7]. Multidrug resistance (MDR) aggravates disease control by intensifying the possibility of spreading resistant pathogens, thus decreasing the efficacy of treatment and creating major economic and health concerns in livestock industries and human healthcare [8]. The MDR, mostly composed of Gram-positive bacteria [9, 10], refers to AMR bacterial strains resistant to at least three different antimicrobial classes. Methicillin-resistant *S. aureus* (MRSA) causes many life-threatening systemic diseases with a high rate of morbidity and mortality worldwide [11, 12]. Vancomycin is commonly used to treat infections caused by MRSA; however, the recent emergence of *S. aureus* infections with high-level resistance to vancomycin [13]. Vancomycin-resistant *S. aureus* (VRSA) has become a serious public health challenge worldwide [12, 14, 15]. Vancomycin-resistant enterococci (VRE) also cause a wide range of illnesses, mostly among patients in hospitals or other healthcare settings, including bloodstream infections, surgical site infections, and urinary tract infections [16, 17]. VRE remains a major threat; consequently, there is tremendous interest in developing novel drugs with bactericidal activity against VRE [18]. Although recent studies by Camsing *et al*. [19] and Somrup *et al*. [20] have reported the presence of AMR for mastitis bacteria in Thailand, however, no reports have described MRSA, VRSA, or VRE in bovine mastitis. Moreover, previous studies have identified these critical AMR genes from several sources of samples from other species, including pigs [21, 22], companion animals [23], and buffaloes [24].

Several studies have reported the prevalence of antimicrobial-resistant bacteria, including MRSA, VRSA, and VRE in companion animals [23] and dairy animals [24–26] across various countries. However, data on these serious antimicrobial-resistant bacteria in bovine mastitis in Southeast Asia, especially Thailand and Cambodia, are very limited and require further investigation. Therefore, this study aimed to detect the genes of MRSA (*mecA*), VRSA (*vanA*), and VRE (*vanB*) in smallholder dairy farms located in Thailand and Cambodia, where farmers have close contact with cows.

## MATERIALS AND METHODS

### Ethical approval

Ethical approval was not required for this study because it did not involve animal samples. All isolates were obtained from the culture collections of the Faculty of Veterinary Medicine, Chiang Mai University, Thailand.

### Study period and location

The study was conducted from July 2023, to March 2024 at the Central Laboratory of the Faculty of Veterinary Medicine, Chiang Mai University, Thailand.

### Bacteria source

**Figure 1:**
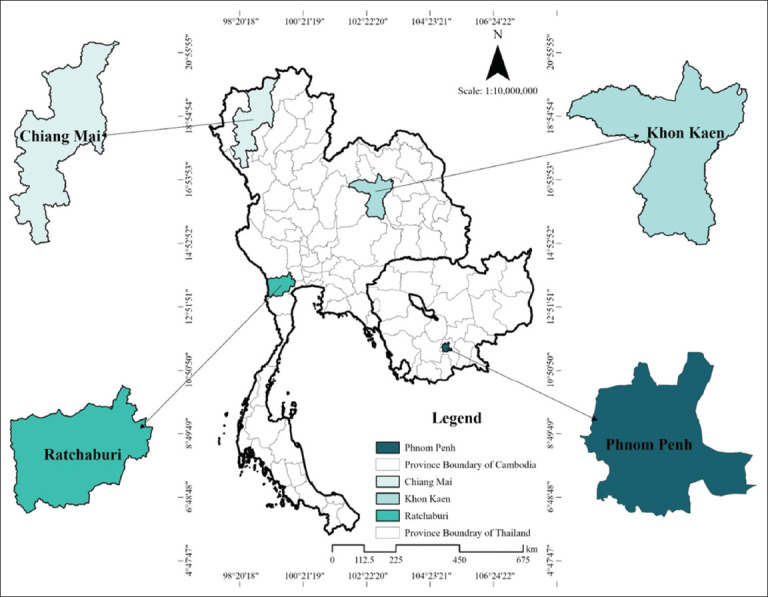
Sampling regions from both countries involved in this study [Source: The map was generated using QGIS v3.34].

A standard *S. aureus*, *S. aureus* ATCC 25923, serving as the control, and 65 bacterial isolates from mastitis samples, including 55 samples of *S. aureus* and 10 samples of *Enterococcus faecium* previously confirmed by MADITOF-MS from −80°C stored in the Faculty of Veterinary Medicine, Chiang Mai University, Thailand, were used. All samples were obtained from subclinical and clinical mastitis milk from various farms in Thailand and two dairy farms in Cambodia, as the management of the two countries differ. The dairy industry in Thailand has been in operation for more than 50 years, while Cambodia is at the beginning of its growth. For *S. aureus*, all samples were obtained from Thailand, including Chiang Mai, a northern province (n = 14), and Ratchaburi, a central province (n = 41). Samples of *E. faecium* were obtained from Khon Kaen, a northeastern province of Thailand (n = 6), and Phnom Penh, Cambodia (n = 4), as shown in Figure 1. All strains were recovered in Tryptic Soya broth (HiMedia, Mumbai, India) and grown overnight at 37°C. The inocula were then cultured on 5% bovine blood agar (HiMedia) at 37°C for 24 h. Isolates were confirmed as *S. aureus* and *E. faecium* by polymerase chain reaction (PCR) amplification of species-specific parts of the 16S rRNA gene and the 23S rRNA gene.

### Antibiotic sensitivity test

According to Clinical and Laboratory Standards Institute CLSI [27], the antibiotic sensitivity test was the disk diffusion method. All colonies were transferred into the normal saline solution to obtain turbidity equivalent to 0.5 McFarland standard [27]. The inoculum was spread onto Mueller–Hinton Agar (Himedia) and placed 15 antibiotic sensitivity disks (Oxoid™, UK): tetracycline (30 μg), ciprofloxacin (5 μg), enrofloxacin (5 μg), gentamycin (10 μg), sulfamethoxazole/trimethoprim (30 μg), cefoxitin (30 μg), chloramphenicol (30 μg), rifampicin (5 μg), oxacillin (1 μg), linezolid (25 μg), penicillin (10 μg), teicoplanin (30 μg), erythromycin (15 μg), nitrofurantoin (30 μg), and vancomycin (30 μg) and then cultured at 37°C for 24 h. The diameters of the inhibition zones were measured and interpreted according to the guidelines of the CLSI [27]. MDR refers to AMR bacterial strains that were resistant to at least three different antimicrobial classes. All isolates of *S. aureus* or *E. faecium* resistant to oxacillin and vancomycin were presumptively identified as MRSA, VRSA, and VRE, respectively. All isolates were additionally identified for *mecA, vanA*, and *vanB* resistance genes.

### Identification of AMR genes: *mecA, vanA,* and *vanB*

Bacterial DNA was extracted using a genomic DNA isolation kit (PureDirex, Bio-Helix, Taiwan) according to the manufacturer’s instructions. The qualities (A260/A280) and concentrations of the extracted DNA were quantified using a NanoDrop spectrophotometer (NanoDrop One, Thermo-Scientific, USA). The extracted DNA was stored at −20°C for further use.

#### Detection of mecA in MRSA

The *mecA* gene was amplified using specific oligonucleotide primers. Both forward and reverse primers ‘5-GTAGAAATGACTGAACGTCCGATAA-3’ and ‘5-CCAATTCCACATTGTTTCGGTCTAA-3’ (band size of 310 bp) [28] and positive control were used (code: MRSA-DMST-206552). A total volume of 50 μL PCR mixture was prepared, consisting of 5 μL of prepared DNA, 25 μL Taq Red Mix (Meridian, Bioscience) with 1× PCR buffer, 0.5 μL of each primer at 20 μmol/L, and 19 μL of nuclease-free water. The thermal cycling protocol for PCR comprised 94°C for 4 min, followed by 30 cycles of 94°C for 45 s, 59°C for 45 s, and 72°C for 1 min, with a final extension at 72°C for 2 min. The amplified products were visualized by electrophoresis in 1.5% agarose gels stained with red safe and captured using a gel documentation system (GelMax Imager, USA).

#### Detection of vanA in VRSA and vanB in VRE

*VanA* was detected using specific oligonucleotide primers: 5′-GGCAAGTCAGGTGAAGATG -3′, and Reverse, 5′- ATCAAGCGGTCAATCAGTTC -3′ for *the vanA* gene (band size 713 bp) [29]. The DNA template used 5 μL, 0.5 μL of each forward and reverse primer, 25 μL Taq Red Mix, and 19 μL nuclease-free water, equal to 50 μL of total volume. The following cycling conditions were followed: initial denaturation at 94°C for 5 min, followed by 40 cycles of 1 min at 94°C, 58°C for 1 min of annealing, 72°C for 2 min, and 5 min at 72°C for the final extension. Meanwhile, specific oligonucleotide primers of *vanB* were done using F-5‘-ATGGGAAGCCGATAGTC-3’ and R-5’GATTTCGTTCCTCGACC-3’, and gene (band size 635 bp) [30]. The DNA template was prepared using 5 μL of template, 0.5 μL of both forward and reverse primers, 25 μL of Taq Red Mix, and 19 μL of nuclease-free water, resulting in a total volume of 50 μL. The protocol conditions for *vanB*: 95°C for 5 min: initial denaturation, 30 cycles and 1 min at 95°C of denaturation, annealing 56°C for 1 min, 72°C for 1 min of extension, and final extension at 72°C for 1 min. The PCR-amplified products were analyzed by electrophoresis on a 1.5% agarose gel and stained with red safe, and images were captured using a gel documentation system (GelMax Imager, USA).

### Statistical analysis

Descriptive statistics were employed to summarize the data, including frequencies and percentages for antibiotic susceptibility and resistance profiles. The antimicrobial resistance patterns were further analyzed using Chi-square tests or Fisher’s exact test for categorical data comparisons, such as resistance rates between bacterial species (*S. aureus vs. E. faecium*) and geographic regions (Thailand vs. Cambodia). Logistic regression models were utilized to identify significant predictors of multidrug resistance (MDR), including bacterial type and sampling region.

Phenotypic and genotypic correlations were assessed using Pearson or Spearman correlation coefficients to determine associations between resistance phenotypes and the absence of resistance genes (*mecA, vanA, vanB*). Graphical representations, including heatmaps and bar plots, were used to visualize the distribution and patterns of resistance. All statistical analyses were conducted using SAS University Edition with a significance level set at p < 0.05.

## RESULTS

### Susceptibility test of *S. aureus* and *E. faecium*

The results of the disk diffusion susceptibility tests are presented in Table 1. All isolates were susceptible to enrofloxacin, trimethoprim-sulfate, cefoxitin, chloramphenicol, rifampicin, oxacillin, and linezolid. Of the 55 *S. aureus* isolates, 65.5% (n = 36) were susceptible to all 12 antibiotics. Most of the bacterial strains showed resistance to penicillin (21.8%) and tetracycline (9.1%). No *S. aureus* strain resisted oxacillin, but 3 strains (SA15, SA16, and SA20) resisted vancomycin. Three strains of *S. aureus* (SA13, SA34, and SA50) resisted tetracycline-gentamycin-penicillin and were identified as MDR bacteria.

**Table 1:** Antibiotic susceptibility tests for *S. aureus* and *E. faecium* that cause mastitis in dairy cattle in Thailand and Cambodia.

Antibiotic	*S. aureus* (n = 55)			*E. faecium* (n = 10)		
	
	S	I	R	S	I	R
	
	n (%)	n (%)	n (%)	n (%)	n (%)	n (%)
Penicillin	40 (72.7)	3 (5.5)	12 (21.8)	-	1 (10)	9 (90)
Vancomycin	49 (89.1)	3 (5.5)	3 (5.5)	10 (100)	-	-
Tetracycline	50 (90.9)	-	5 (9.1)	-	-	10 (100)
Gentamicin	51 (92.7)	-	4 (7.3)			
Ciprofloxacin	54 (98.2)	1 (1.8)	-	8 (80)	2 (20)	-
Enrofloxacin	55 (100)	-	-			
Trimeth/Sulfame*	55 (100)	-	-			
Cefoxitin	55 (100)	-	-			
Chloramphenicol	55 (100)	-	-	9 (90)	1 (10)	-
Rifampicin	55 (100)	-	-	-	4 (40)	6 (60)
Oxacillin	55 (100)	-	-			
Linezolid	55 (100)	-	-	10 (100)		
Teicoplanin				10 (100)	-	-
Erythromycin				-	8 (80)	2 (20)
Nitrofurantoin				10 (100)	-	-

* Trimeth/Sulfame=Trimethoprim-sulfamethoxazole.

*S. aureus*=*Staphylococcus aureus*, *Enterococcus faecium*=*Enterococcus faecium,* S=Susceptible, I=Intermediate, R=Resistant

In contrast to *S. aureus*, *E. faecium* had high levels of antibiotic resistance, including tetracycline (100%), penicillin (90%), rifampicin (60%), and erythromycin (20%), and all these antibiotics were not susceptible for *E. faecium*. *E. faecium* showed susceptibility (100%) to antibiotics vancomycin, linezolid, teicoplanin, and nitrofurantoin. All strains of *E. faecium* resisted 1, 2, 3, and 4 antibiotics at concentrations of 10%, 30%, 40%, and 20%, respectively. All six *E. faecium* isolates from Khon Kaen province, Thailand, were classified as MDR bacteria. In contrast, the four strains of *E. faecium* isolated from a dairy farm in Phnom Penh, Cambodia, resisted only tetracycline and penicillin. The six MDR *E. faecium* strains found in this study resisted tetracycline-penicillin-rifampicin (n = 4: EF1, EF2, EF5, and EF6) and tetracycline-penicillin-rifampicin-erythromycin (n = 2: EF3 and EF4). No vancomycin resistance was detected in *E. faecium*.

**Figure 2:**
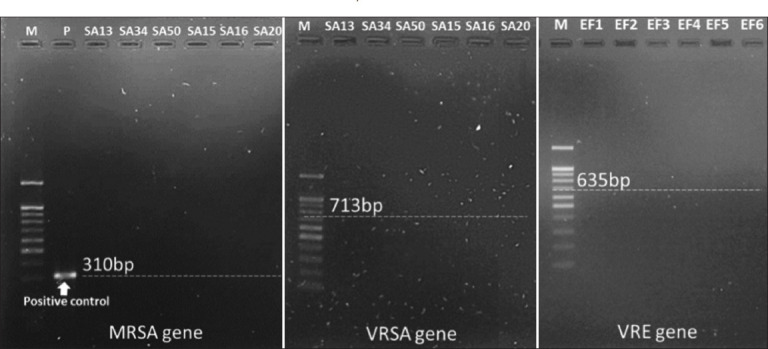
Identification of MRSA (*mecA*) and VRSA (*vanA*) genes for *Staphylococcus* *aureus* with multidrug-resistant (SA13, SA34, and SA50) or vancomycin-resistant (SA15, SA16, and SA20) and VRE gene (*vanB*) for *Enterococcus faecium* with multidrug-resistant (EF1-EF6). MRSA=Methicillin-resistant *Staphylococcus aureus*, VRSA=Vancomycin-resistant *Staphylococcus aureus*, VRE=Vancomycin-resistant enterococci.

All *S. aureus* strains were tested for *mecA* and *vanA*, but all tested negative for either gene. Figure 2 shows negative results for MRSA and VRSA genes in both MDR *S. aureus* and vancomycin-resistant *S. aureus* strains. In addition, no *van B* gene was detected in *E. faecium*. Figure 2 shows the negative VRE gene in MDR *E. faecium*.

## DISCUSSION

In this study, we included samples from both Thailand and Cambodia to identify MRSA, VRSA, and VRE in dairy farms in this area, where most farms are smallholder dairy farms with close contact between humans and animals, providing insights into the potential risk of transmission and contamination of these resistant genes among cattle, humans, and the environment within the farms. Most antibiotics tested were effective against *S. aureus*, with 65.5% of isolates from bovine mastitis showing full susceptibility, including enrofloxacin, trimethoprim-sulfamethoxazole, cefoxitin, chloramphenicol, rifampicin, oxacillin, and linezolid (Table 1). In support of a recent study from northern Thailand, 100% susceptibility to cefoxitin was also reported [19]; this showed that many strains remain treatable with conventional antibiotics in Thailand. In contrast, the highest susceptibility of *S. aureus* isolates to antimicrobials was observed with ciprofloxacin (82.90%), followed by cefotaxime, gentamycin, vancomycin, nitrofurantoin, tetracycline, and co-trimoxazole (51.20%) [31, 32]. Our notable resistance to penicillin (21.8%) and tetracycline (9.1%) was in line with global trends, where antibiotic resistance is widespread due to its overuse in veterinary medicine. This finding is consistent with a previous study by Rao *et al*. [33], showing that the isolates of *S aureus* were most resistant to penicillin (42 % in dairy cattle). A previous report in Ukraine shows that the isolates of *S. aureus* were highly resistant to tetracycline (21.4%) [34]. However, 35% (19/55, including 15 resistance and four intermediate) of *S. aureus* were not susceptible to all antibiotics in this study, and three of them were MDR, which might be a warning for careful antibiotic usage. Previously, a strain of streptococci and staphylococci other than *S. aureus* were reported as MDR mastitis pathogens in Thailand [28]. Unfortunately, there are no reports on the MDR of *S. aureus* in animals in Cambodia. This is the first report of MDR *S. aureus* in Thailand. The MDR *S. aureus i*s a widespread multi-host infectious bacterium. Recently, MDR *S aureus* has been associated with subclinical mastitis in dairy cows and is considered an emerging zoonotic pathogen [35].

Despite the relatively small number of *E. faecium* isolates, a high percentage of AMR was observed, with all isolates showing resistance to tetracycline. A similar case of highly resistant *E. faecium* was reported by Yang *et al*. [36], who investigated AMR in *E. faecalis* from bovine mastitis in China and found high resistance rates to tetracycline (87.7%) and erythromycin (79%). This study found that 90% and 60% of *E. faecium* isolates were resistant to penicillin and rifampicin, respectively. These findings are consistent with those of Paschoalini *et al*. [26], who reported a high resistance rate to penicillin and rifampicin in *E. faecium* from milk samples. Consequently, of the high AMR in *E. faecium*, 60% (6/10) of MDR, including resisted three-antibiotic-groups isolates (n = 4) and resisted four-antibiotic-groups isolates (n = 2), were observed in this study. MDR enterococci are highly prevalent mastitis pathogens in China, ranging from 21% to 26% [37]. The evolution of AMR in these organisms affected patients with severe infections caused by MDR *E. faecium* worldwide, resulting in a significant decrease in therapeutic options [37, 38].

Based on antibacterial susceptibility tests, MRSA was not detected in any of the *S. aureus* strains. However, this study identified three phenotypic VRSA strains, indicating that AMR issues were particularly concerning. Subsequently, no *mecA* was detected; however, no *vanA* was detected in the phenotypic VRSA isolates in this study. Similarly, a previous study by CLSI [27] identified no antibiotic-resistance genes for all Staphylococci in Thailand. The evidence that some phenotypic MRSA strains do not carry *mecA* has been reported [39]. This finding is consistent with other studies by Bignardi *et al*. [40] and Moon *et al*. [41], which reported similar discrepancies between the phenotypic resistance and genotypic profiles of MRSA. The possibility that this result could be linked to the relatively small number of isolates in our study was considered. This limitation may affect the detection and representation of genetic diversity associated with methicillin and vancomycin resistance. According to the phenotype, no *vanB* was found in *E. faecium*. In general, various enterococcal species exhibit various degrees of resistance to vancomycin and *vanB* gene expression [42]. These findings provide clear evidence that mechanisms other than the presence of *vanA* gene are responsible for VRSA and that molecular methods alone are not sufficient for the confirmed characterization of VRSA isolates. Therefore, both phenotypic and genotypic factors should be considered to confirm VRSA characterization.

## CONCLUSION

This study provides critical insights into the AMR patterns of *S. aureus* and *E. faecium* isolates cause bovine mastitis in Thailand and Cambodia. Notably, *S. aureus* exhibited resistance to key antibiotics such as penicillin (21.8%), tetracycline (9.1%), and gentamycin (7.3%), with three isolates identified as MDR. In contrast, *E. faecium* showed 100% resistance to tetracycline and 90% to penicillin, with 60% classified as MDR. Although VRSA strains were identified phenotypically, no *mecA, vanA*, or *vanB* genes were detected. These findings highlight the significant threat posed by MDR pathogens in dairy farming and underline the complexity of AMR mechanisms.

The study’s comprehensive approach, combining phenotypic and genotypic analyses, ensures a thorough investigation of AMR. By including isolates from Thailand and Cambodia, it provides a comparative perspective and highlights differences in agricultural and veterinary practices. Focusing on *S. aureus* and *E. faecium*, pathogens of significant clinical and public health relevance, enhances the study’s impact.

However, the relatively small sample size and limited geographical representation may restrict the generalizability of the findings. Isolates from a few dairy farms may not capture the full variability in resistance patterns across the region. In addition, the inability to detect *mecA, vanA*, and *vanB* in phenotypically resistant strains suggests the need for more advanced genotyping techniques to uncover alternative resistance mechanisms.

Future studies should include larger sample sizes and more diverse geographic coverage to validate these findings and enhance their applicability. Advanced molecular techniques, such as whole genome sequencing, could be employed to identify novel or alternative AMR mechanisms. Interventional studies investigating the impact of antibiotic stewardship programs and alternative treatments like probiotics or phage therapy could provide actionable solutions to combat AMR in dairy farming. A One Health perspective assessing the transmission dynamics of AMR between animals, humans, and the environment within dairy farms would also offer valuable insights into mitigation strategies.

This study underscores the urgent need for prudent antibiotic use and robust surveillance systems in dairy farming to mitigate the rising threat of AMR. Addressing the identified limitations and pursuing future directions will be crucial for preserving the efficacy of antibiotics and ensuring the sustainability of livestock farming practices.

## AUTHORS’ CONTRIBUTIONS

SN: Sample collection, study and laboratory work, and drafted the manuscript. MI: Study design, planning, laboratory work, and revised the manuscript. AS and WC: Planned the study and revised the manuscript. WS: Study design and execution and revised the manuscript. All authors have read and approved the final manuscript.
